# Phenols in Leaves and Bark of *Fagus sylvatica* as Determinants of Insect Occurrences

**DOI:** 10.3390/ijms12052769

**Published:** 2011-04-26

**Authors:** Panos V. Petrakis, Kostas Spanos, Alan Feest, Evangelia Daskalakou

**Affiliations:** 1 Laboratory of Entomology, National Agricultural Research Foundation, Institute for Mediterranean Forest Ecosystem Research, Terma Alkmanos, Athens 11528, Greece; E-Mail: edaskalakou@fria.gr; 2 National Agricultural Research Foundation, Forest Research Institute, Vassilika 57006, Thessaloniki, Greece; E-Mail: kspanos@fri.gr; 3 Water and Management Research Centre, Faculty of Engineering, University of Bristol, Bristol BS8 1TR, UK; E-Mail: a.feest@bristol.ac.uk; 4 Ecosulis Ltd., The Rickyard, Newton St. Loe, Bath, BA2 9BT, UK

**Keywords:** *Fagus sylvatica*, entomofauna, phenolics, secondary plant chemistry, insect traps, clustering, discriminant analysis

## Abstract

Beech forests play an important role in temperate and north Mediterranean ecosystems in Greece since they occupy infertile montane soils. In the last glacial maximum, *Fagus sylvatica* (beech) was confined to Southern Europe where it was dominant and in the last thousand years has expanded its range to dominate central Europe. We sampled four different beech forest types. We found 298 insect species associated with beech trees and dead beech wood. While *F. sylvatica* and *Quercus* (oak) are confamilial, there are great differences in richness of the associated entomofauna. Insect species that inhabit beech forests are less than one fifth of those species living in oak dominated forests despite the fact that beech is the most abundant central and north European tree. There is a distinct paucity of monophagous species on beech trees and most insect species are shared between co-occurring deciduous tree species and beech. This lack of species is attributed to the vegetation history and secondary plant chemistry. Bark and leaf biophenols from beech indicate that differences in plant secondary metabolites may be responsible for the differences in the richness of entomofauna in communities dominated by beech and other deciduous trees.

## 1. Introduction

In beech forests, beech trees have possibly the highest amount of phenolics compared to co-occurring species: in the leaves [1], bark [2], wood [3], and roots [4]. Phenolics inhibit feeding by various fungi [5] and potential herbivores except specialized ones capable in detoxifying phenolics such as *Cryptococcus fagisuga* [2,6,7]). Dead wood insects rely on the fungi associated with wood so do not normally colonize beech unless they are able to avoid the phenolic rich tissues. Members of the family Aradidae (Heteroptera) are an example of such insects. Other insects, especially those belonging to Coleopteran families Cerambycidae and Buprestidae, colonize beech trees after the phenolic content has reduced.

In the context of our investigations of the entomofauna of beech forests in Greece, we present the first inventory of the insects collected in four beech forest types in central and northern Greece. We also present estimates of the phenolic content of bark and leaves of beech trees in these sites and we discuss whether and to what degree it determines insect richness.

## 2. Materials and Methods

### 2.1. Sites Studied

**Figure 1.**
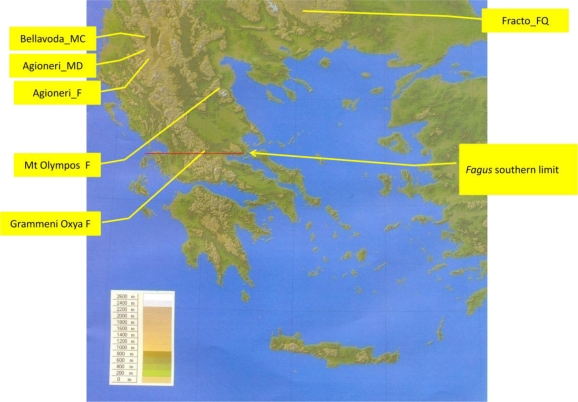
Map showing the sampled sites in Greece. The southern limit of *F. sylvatica* is also shown.

Four sites were intensively studied (insects collected, bark and leaf phenolics determined and plant coverage measured) in northern Greece (Figure 1). The sites were on a variety of geological substrates and deep soils. Two sites were selected at (i) Aghioneri, Prespa, Mt Triklarion, 40°44′N, 21°7′E, northern exposure, 1650 m a.s.l., is a monospecific 120 year old forest; (ii) Aghioneri, Mt Triclarion, 40°43′N, 21°8′E, north-western exposure, 1600 m a.s.l., is a mixed deciduous 60 year old forest with several other species (Table 1); (iii) in the virgin forest at Fracto, Drama Mt Western Rodopi, 41′33′N, 24°31′E, northern exposure, 1600 m a.s.l., where beech co-dominates with *Quercus frainetto* and *Q. petraea*; (iv) Bellavoda, Prespa Mt Peristeri, 40°50′N, 21°13′E, northern exposure, 1700 m a.s.l., where beech co-dominates with *Abies borisii-regis* [8].

In each site, at a relatively natural spot, a basic square plot (1 ha area) was established, and plant coverage was measured in nine quadratic subdivisions (1/10 hectare sub-plots) with a photographic technique to estimate the amount of the height profile of the foliage [9,10]. All values were then averaged and expressed as percentage coverage, which as a rule sum up to values greater than 100% for each plant species.

### 2.2. Insect Sampling

Insects were sampled at each location within the basic plot used for the estimation of plant coverage, by six methods in the period May–August: (i) twenty pitfall traps uniformly spread in the basic plot and baited with tuna meat in ethylene glycol and ethanol (5:1) sampled the epiedaphic fauna, and the contents were collected every week; (ii) five Malaise traps were used once a month in the period May–July for all actively flying insects and cerambycids-buprestids for 6 h a day (900–1500 h); (iii) ten permanent window intercept traps were also hung from the trunks of five beech trees for the same purpose; (iv) Sweep netting up to 3 m height for the collection of foliage dwelling insects and hand collecting on the tree trunks and branches was used for all insects in the lower foliage and vegetation; (v) dead wood (fallen branches with diameter >7 cm) was sampled *in situ* by hand collecting. Nine branches with diameter >7 cm, in each basic plot, were placed in epilectic traps left on the ground and examined weekly for two months; (vi) chemical knockdown using *C*-permethrin (1:50) and mistblowing in the crown [11] was used for five trees at the center of the basic plot and the four corner sub-plots, in still air conditions (these were the five individual beech trees from which bark samples were taken (see below). The falling insects were collected in a piece of plasticized cloth spread underneath each tree to cover the entire crown and held at 0.5–1 m height by means of wooden rods to avoid interference from understorey vegetation. The collection of insects was made from the cloth twice, 30 min and 2 h after mistblowing ([12,13]). All insect catches were pooled to represent the insect species of the beech forest stand.

All insects were temporarily stored in 80% ethanol, transferred to the laboratory, and identified to morphospecies level. Identification of the insect material is an ongoing process. Voucher insect specimens have been deposited in the Entomological Collection in the Institute for Mediterranean Forest Ecosystem Research (IMFE), Athens, Greece.

### 2.3. Plant Material Sampling and Phenolic Content Estimation

In each site, five *F. sylvatica* trees were selected at the central area of the basic plot and the four corner subplots to capture the variability in the trees and their typical features in the area of the plot. In June, when the majority of the plant species were in full growth approximately 50 g of bark, both the inner and the outer layers from three sides of the trunk at breast height and 120° angular distance, was removed, brush cleaned, and put in dry-ice (CO_2_). Following standard analytical procedures [2,14], we freeze dried, ground, and extracted the phenolic content of 40 g of bark (5 × 100 mL MeOH) for 24 h at room temperature (∼25 °C).

HPLC grade standards were purchased from Aldrich (Greece) and Biobiopha Co., Ltd (China) or obtained from the laboratories of V. Roussis (University of Athens) (catechin, syringin), C. Vagias (University of Athens) (glucodistylin, taxifolin-xylopyranoside), M. Kouladis (University of Athens) (quercetin, coniferin, isoconiferin), Ph. Dais (University of Crete, Irakleio, Crete) (apigenin, chlorogenic acid, luteolin). Standard compounds were used as internal standards and also as identification means of the compounds in the chromatogram from the elution time.

From the extract we estimated the concentration of each compound in a HPLC machine by using the C18 reverse phase column (3 μm, 200 × 4.6 mm i.d., 200 m^2^/g) (Spherisorb, Waters) and a water/methanol gradient with the detection at 254 nm. The elution gradient consisted of an initial 80% 0.01 M H_3_PO_4_ for 3 min, and a linear gradient from 20% to 100% MeOH for 60 min at a flow rate of 0.8 mL·min^−1^. After each analysis and before the next, the column was washed with 100% MeOH for 3 min and returned to 20% MeOH and re-equilibrated for 10 min. Quantification was done by means of internal standards and the concentrations were expressed in mg/g (bark dry weight).

The leaf polyphenol concentration was measured at each site in mid-July on the same five trees as above. From these trees 100 leaves were sampled in each of the following categories: (i) leaves, infested and non-infested by herbivorous insects as judged from visible damage marks. Leaves with just one feeding sign, which was presumably a feeding trial, were categorized as non-infested; (ii) small and large leaves judged by their position on the twig; small leaves are usually at the distal end of twigs and large leaves at the proximal end; (iii) shaded and un-shaded leaves respectively located at the lower parts and the top of the tree crown. The air-dried leaf samples were ground and the coarse powder passed through a 250 μm mesh size screen. Approximately 100 mg of leaf fine powder was analyzed according to the modified Folin-Ciocalteu method ([15,16] Graca and Bärlocher, 1998; Bärlocher and Graca, 2005). The concentration of polyphenols was measured in a spectrophotometer at 760 nm. Absorbances were converted to concentrations on a tannin based calibration curve and expressed in mg/g (dry leaf weight).

### 2.4. Data Analysis

Relationships between sites on the basis of the coverage of plant species were analyzed by hierarchical clustering of Euclidean distances of sites and the joining algorithm of minimum variance (Ward joining method). The same site sets were used as a basis for the hierarchical joining of Euclidean distances but substituting the phenolic concentrations of beech trunk bark and leaves in each site served as site descriptors. Classification hierarchies were compared with the insect species richness of each site on the basis of the entire hierarchical topology. Canonical discriminant analysis (CDA) [17] in a stepwise forward mode was applied to beech trees in the four sites described according to individual tree (bark and leaves) phenolic concentration. For the manipulation of data and the application of the classification methods we used the packages “vegan” [18], *R* language [19] and “SYSTAT” [20].

## 3. Results

The four sites yielded a total of 298 insect morphospecies in thirty-seven families in six orders. Ongoing formal identification of several other morphospecies (*ca*. 47) belonging to Hymenoptera (parasitica and acuelata, except ants), Scarabaeoidea, and Orthoptera is not expected to change basic patterns since they represent the species already identified. Carabidae and Hemiptera are identified to species level. Other beech forest soil invertebrates were also collected in pitfall traps but are not considered further here since they indicate soil types rather than beech tree relations.

**Figure 2.**
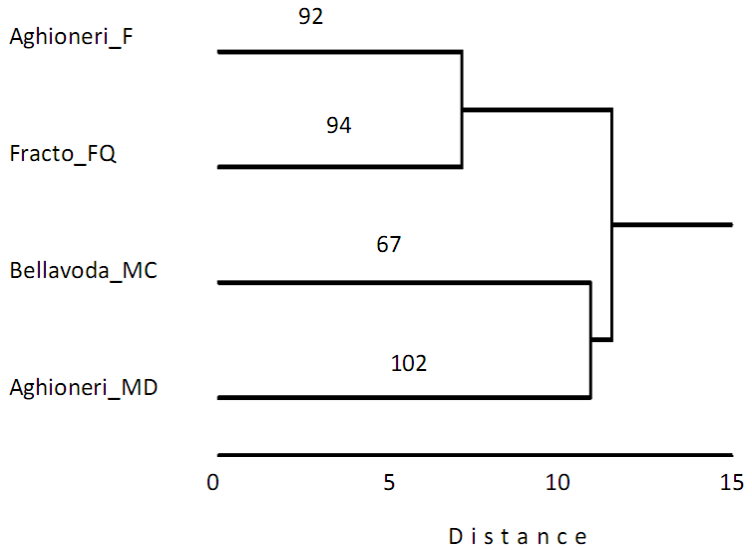
Dendrogram of the hierarchical classification of four beech forest types in Greece on the basis of co-dominant and sub-dominant tree-shrub species. The linkage algorithm is the Ward minimum variance and the distance metric is the Orloci’s chord distance. The numbers above branches show the number of insect species (morphospecies level).

**Figure 3.**
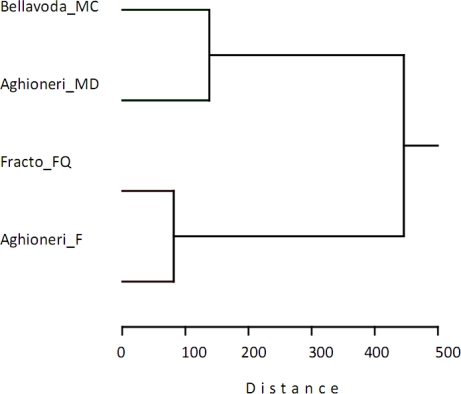
Dendrogram resulting from the hierarchical classification of the main beech forest types in Greece. The linkage algorithm is the Ward minimum variance and the distance metric is the Euclidean distance on the basis of the phenolic content of the bark.

The hierarchical classification of sites on the basis of plant cover is shown in Figure 2. The same site clustering occurred using the phenolic content and the results are shown in Figure 3. The two groups both recognized a split of the Aghioneri sites in two main branches. The monospecific beech site (Aghioneri_F 92 insect species) is grouped with Fracto (94 insect species). The Aghioneri site (Aghioneri_MD 102 insect species) containing the mixed deciduous forest is grouped with the mixed with fir trees site on Bellavoda (67 insect species). The insect species richness is not consistent with this grouping since both poorest and richest sites belong to the same group. In addition the grouping does not reflect geographical proximity.

**Figure 4.**
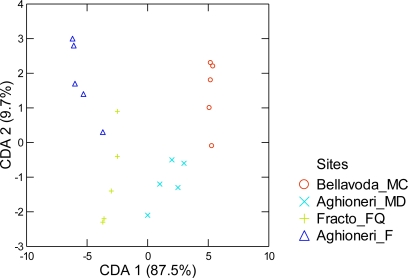
Diagram of canonical discriminant analysis of the beech trees grouped according to the site and described in individual phenolic concentrations. Two discriminant axes account for a significant percentage (97.3%) of variation in the original data [17].

In the plane of the discriminant analysis (*λ*_Wilks_ = 0.004; *F*_approximation_ = 19.65; *df*_1_ = 12; *df*_2_ = 34; *p* < 10^−4^) (Figure 4), which accounts for 100% of the variation in phenolic concentration, the percentage variation accounted for by each axis (87.5%, 9.7%, 2.7%) makes the first axis (CDA-1) dominant. The first discriminant axis separates the same groups of Figure 2 and 3 while the second axis separates individual sites. It can be seen also that sites at Aghioneri are located along the main diagonal of the CDA principal plane. This diagonal reflects a combined gradient of plant species richness.

Seven phenolic compounds were found in the beech tree bark in all sites (Table 2) and all of them were also found in another study on European beeches [2] (Dübeler *et al*., 1997). Three groups of phenolics can be created according to their concentration ranking.

The first group consists of isoconiferin and syringin which attain the highest concentrations. The second group consists of phenolics having intermediate concentrations. The third group consists of compounds with consistently low concentrations. In CDA the most informative phenolics belong to all three groups (Table 3). Two phenolics (syringin and catechin) are responsible for the separation of sites in Figure 4 yet they are found in low and high concentrations in all sites. The other two compounds (coniferin and *R*-glucodistylin) can be found in intermediate and low concentrations while taxifolin-xylopyranoside is found in intermediate concentrations. The remaining two phenolics, *iso*-coniferin, and *S*-glucodistylin were relatively uninformative with low *F*-values (1.86, 2.66 and 1.62, respectively. Not shown in Table 3) and varied (lowest to highest) in their concentrations.

The classification ability of CDA is shown in the classification table (Table 3). All sites were successfully classified (100%) on the basis of phenolics. However, the ability of beech trees to predict the site origin of other syntopic trees is somewhat restricted (90%).

**Figure 5.**
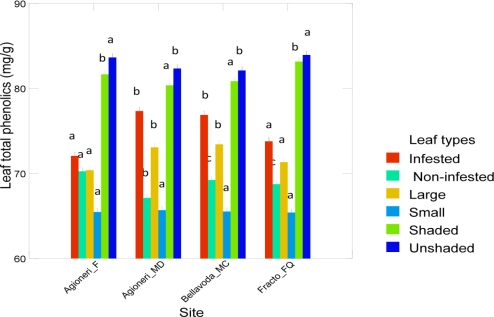
Diagram showing the mean content of total phenols (mg/g) in the six leaf categories for each site. Bars with the same letter on top are not significantly different across sites *i.e.*, those having the same color at *p* = 0.01.

The number of insects is expected to depend inversely on the concentration of phenolics [21]. Since phenolic compounds have fluctuating concentrations as precursors of lignin and provide defense mechanisms against insects and fungi [2,5,21], we used the bark and leaf phenolic sum content at each site. An interaction term (*site*) × (*phenol content*) was also added to the regression model: (*number of insect species*) = *ct* + (*phenolic content*) + (*site*). It was found that the number of insect species depends on site effects (*F* = 5.34; *df*_1_ = 3; *df*_2_ = 12; *p* = 0.01) but both the effect of phenolics and the interaction (in a mathematical sense) of each site with the phenolic concentration of beech bark were insignificant. If only leaf categories are taken into account then the phenolic content of leaves is significantly affected by the insect damage, the size and the position of the leaves on the crown (Table 2) (*N* = 120; *r* = 0.98; *F* = 980.23; *df*_1_ = 5; *df*_2_ = 96; *p* < 10^−4^) in each site (*F* = 2.93; *df*_1_ = 3; *df*_2_ = 96; *p* = 0.0035) and the combination of them (*F* = 11.89; *df*_1_ = 15; *df*_2_ = 96; *p* < 10^−4^). The variation of total leaf phenolics in all categories across sites is given in Figure 5. Without regard to sites but to the groups of Figure 3 the phenolic concentration was highly positively correlated to the number of insects species (*N* = 20; *r* = 0.92; *F* = 14.31; *df*_1_ = 1; *df*_2_ = 17; *p* = 0.001). If no grouping was taken into consideration then the regression: (*number of insect species*) = *ct* + *b* (*total phenolic content*) was significant but the correlation coefficient was positive but low, and the regression coefficient was practically nil (*r* = 0.27; *ct* = 118.0; *t_ct_* = 11.09; *b* = −0.008; *t_b_* = −2.81; *p* = 0.01).

## 4. Discussion

Phenolics are considered to be powerful defenses against fungi [5,21–23] and insect herbivory [2,24–26] and they are engaged in the allelopathic interactions with other plants [26,27]. Methods for the extraction, quantification and chemical characterization of phenolics in beech primarily concern the coccid *Cryptococcus fagisuga* that attacks north-American and European populations of beech [2,6] and methods have been developed to deal with the phenolic content of beech wood [3] reviewed in Bedgood *et al*. [14]. Different delivery of phenolics and chemical analysis gives divergent results, for an already fluctuating profile of phenolics concentrations, it is essential that the analytical protocol be kept constant throughout the study in all beech forests.

Hierarchical classification gave the same pattern of sites described either for plant coverage or bark and leaf phenolic concentration. However, the species richness of insect entomofauna was very little affected by the phenolic content of bark and leaves (*r* = 0.27) and when the effect of the site is taken into consideration the phenolic effect on insect species richness, considered aggregately as (*site*) × (*phenolic content*) and (*phenolic content*) alone, was insignificant. The hierarchical structure revealed a very high effect on insect species richness (*r* = 0.92). This indicates that beech forest stands harbor insect species in concert with co-occurring plants *i.e.*, a significant site effect through many mechanisms that cannot be attributed to a single factor such as the phenolic content in the bark and leaves. Evidently, the role of phenolics in aiding the trees to cope with attacks from insects and fungi is masked by their ability to increase beech tree durability under harsh climatic conditions [1,21]. The range of variation in phenolic content is not an obstacle since the sampling for phenolics took place in June when the peak of bark phenolics is observed [2] and the leaves have more or less completed growth.

The fact that individual phenolics vary in quantity in different stands may be due to the fact that some of them are precursors of lignin biosynthesis pathway (e.g., coniferin) and consequently their concentration may depend on the progress of this biosynthetic pathway. Thus, the it can be inferred that the defensive function of phenolics against insects may be a secondary result of the primary process of lignin biosynthesis. For example, the CDA informative compounds coniferin and syringin have reduced concentrations in the new wood formation resulting from traumatic periderm formation isolating infected tissue [24,25,28]. Research on this is rare and almost all existing studies have been conducted in the framework of beech responses to *C. fagisuga* infestation [2,6]. Nevertheless, this response of the beech tree apparently occurs in all cases of insect attack and associated fungi [21].

The relationships of insects with plants in beech forests have also an historical component that complicates the explanation of the patterns revealed in this study. *F. sylvatica* was able to spread quickly from glacial refugia to occupy new territories [29] (Lang, 1994). For instance, the sibling species *F. orientalis*—which was once considered a subspecies of *F. sylvatica*—possesses this property and Southern Europe (North Italy) was occupied by an *F. orientalis* complex during the last interglacial [30]. European beech is the most abundant forest tree in Europe and it would be even more abundant in the current interglacial if there was not a migration lag in its Holocene dispersion [31]. In addition, it is able to: (i) determine the fauna of and the association with other plants in beech forests by allelopathy [27,31,32]; (ii) reduce the numbers of soil arthropods [27]; (iii) enrich soil humus with substances resisting biodegradation; (iv) juvenile stages resists harsh abiotic conditions [33,34]; and (v) it is able to exploit the ecological conditions created by other plants. As a result it grows better in plant rich communities such as the Aghioneri_MD site, [7] (Schmidt and Leuschner, 2009). We found that even in beech dominated forests like the site at Aghioneri_F, it has a constant insect species richness although the phenolic response to insect foliage feeding damage is insignificant (Figure 5).

Communities dominated by *F. sylvatica* conform to the paradigm of a “European crucible” [35]. According to his paradigm there are not many invaders (especially phytophagous insects) in Europe in comparison to North America and Asia due to ecosystem resistance because of natural enemies and competitive superiority of existing taxa. European beech forests exhibit all the properties required by the paradigm. The CDA based on phenolics shows this pattern (Figure 4). The beech sites involving conifers and other deciduous trees are restricted to the positive end of the first CDA axis (87.5%) a fact that supports the idea of a common factor. A comparison of the leaf total polyphenols in *F. sylvatica* (Figure 5) with that of the North American congener *F. grandifolia* [6,36], shows greater values in all leaf categories for *F. sylvatica*.

In a detailed study involving knockdown chemical fogging of seven beech trees co-occurring with *Abies alba*, in the Dinaric mountains, Slovenia [12] it was found that the Heteroptera fauna of tree crowns is dominated, in terms of species richness, by predatory species (43.7%). Among phytophagous species, the species richness of vagile insects (25%) outnumbered purely phytophagous species (12.3%) and insects with mixed diet (12.5%). This pattern was caused by insects that also exploited co-occurring plants.

Beech forests grow at high altitudes (in Greece above 800 masl and also at lower altitudes in northern Greece) [8] where the prevailing climatic conditions are unsuitable for insects for at least some of the year. In spite of this, many insect species were found in all four sites. Speciose insect groups have different ways to confront adverse conditions. Ground beetles usually shelter in the soil litter and in tree crevices where they can find shelter and food [37]; or live in dead wood such as in the Aradidae (5 species). These insects prefer wood infected with certain fungus species [38]. The dead wood of *F. sylvatica* is suitable for fungi that tolerate the phenolic content. In this way fungi can attain a large biomass in beech dead wood aided by the higher humidity of shady places.

This complicates the correlation between the insect species numbers and the phenolic content of beech bark and leaves. It is believed that increased phenolic concentrations reduce herbivory although species richness can be increased as a result of co-evolutionary processes that result in monophagy or oligophagy. Indeed, we found a small but significant positive correlation (*r* = 0.27) and a very low regression coefficient (−0.008), indicating that some insects can tolerate phenolic levels. If the site effect is taken into consideration then this picture is blurred.

It is a paradox that no monophagous species were found in *F. sylvatica*. Nickel [36] found the same in the leafhoppers of beech in Germany. It seems that the same pattern may hold everywhere in Europe. This paradox is intensified by the fact that beech is the most widespread species in Europe [29]; also the thermal properties of beech bark force it to occur in closed stands, often with other tree species, where sunrays cannot overheat the trunk [39] hence its ability to tolerate other tree species [8]. Because of this property many locality and village names e.g., *skotini* (=dark site) indicate the reduced light conditions in beech stands. The con-familial *Quercus* with the same or greater phenolic content harbors several monophagous species [36]. It is possible that the population cycles in the interglacial expansions of beech were prohibitive for monophagy to evolve coupled with the migration lags of the plant. Evidently, the evolution of specificity in insects needs more extensive time periods than those corresponding to the interglacial expansion phases of beech.

## 5. Conclusion

We found that almost three hundred insect species were associated with beech trees and dead beech wood in the study sites. Insect species that inhabit beech forests are less than one fifth of those species living in oak dominated forests despite the fact that beech is the most abundant central and north European tree and *Fagus* and *Quercus* belong to the same family (Fagaceae). There is a distinct paucity of monophagous species on beech trees and most insect species are shared between co-occurring deciduous tree species and beech. This lack of species is attributed to the vegetation history and to a lesser degree to the secondary plant chemistry. Phenolics are engaged in the biosynthesis of lignin which is abundant in trees and their concentrations may reflect the different progress of this biosynthetic pathway among beech trees even of the same population. Also phenolics are found in similar amounts in the bark and leaf of beech and show great fluctuations among trees affecting the harbored entomofauna. Combining this with the astonishing lack of monophagous species on beech it is shown that differences in plant secondary chemistry may be responsible for the differences in the richness of entomofauna of beech trees.

## Figures and Tables

**Table 1.** Sampled sites in Greece and the tree species growing alongside *F. sylvatica*.

***s/n***	**Site and Name Codes**	**Community Type**	**Co-Dominant and Sub-Dominant-Plant Species**
1	Aghioneri, Prespa, Mt Triclarion, Greece; Aghioneri_F	*Fagus sylvatica* monospecific forest	*Fagus sylvatica* (incl. *F. moesiaca*)

2	Aghioneri, Prespa, Mt Triclarion, Greece; Aghioneri_MD	Mixed beech forest with several co-dominant and sub-dominant deciduous tree species	*Fagus sylvatica*, *Quercus frainetto*, *Q. cerris*, *Corylus avellana*, *Acer* spp. (*pseudoplatanus*, *campestre*, *obtusatum*, *tataricum*), *Carpinus betulus*, *C. orientalis*, *Ostrya carpinifolia; Colutea arborescens*, *Fraxinus ornus*, *Juniperus oxydedrus* (sparse)
3	Bellavoda, Prespa, Mt Peristeri, Greece; Bellavoda_mixed beech forest	Mixed beech forest with co-dominance of *Abies borisii regis*	*Fagus sylvatica*, *Abies borisii regis*, *Juniperus oxydedrus*, *Pteridium aquilinum* in the openings
4	Fracto virgin forest, Drama, Greece; Fracto_FQ	Mixed beech forest with oaks	*Fagus sylvatica*, *Quercus frainetto*, *Q. petraea*

**Table 2.** Concentration of biophenols in *F. sylvestris*. The values referring to bark are individual compounds measured by HPLC and the values referred to leaves are total phenolics measured by the modified Folin-Ciocalteu method. All values are expressed as mg/g (dry mass). The numbers are means (std) of five trees.

**Plant Tissue Analyzed**	Compound or Leaf Type Analyzed*	**Bellavoda_MC**	**Aghioneri_MD**	**Fracto_FQ**	**Aghioneri_F**
Bark	catechin	6.5 (0.5)	5.5 (0.7)	5.4 (0.7)	5.1 (1.4)
Bark	*cis*-coniferin	45.2 (5.2)	25.2 (6.0)	17.1 (2.1)	18.3 (2.6)
Bark	*cis*-isoconiferin	61.1 (6.8)	61.5 (9.6)	38.1 (7.2)	36.9 (2.5)
Bark	*cis*-syringin	62.3 (8.1)	50.6 (6.0)	55.3 (15.5)	33.6 (6.7)
Bark	*R*-glucodistylin	8.9 (1.8)	5.4 (0.5)	4.1 (0.4)	3.2 (0.3)
Bark	*S*-glucodistylin	11.1 (1.9)	5.9 (1.4)	8.5 (2.1)	4.9 (0.6)
Bark	taxifolin-xylopyranoside	46.1 (5.4)	37.0 (4.2)	20.4 (2.0)	13.6 (4.4)
Leaves	Non infested	69.2 (1.0)	67.1 (1.2)	68.7 (0.5)	70.3 (0.8)
Leaves	Infested	76.9 (0.7)	77.3 (1.2)	73.8 (1.0)	72.1 (0.9)
Leaves	Small	65.5 (0.9)	65.7 (0.9)	65.4 (1.0)	65.5 (1.2)
Leaves	Large	73.4 (4.0)	73.1 (5.5)	71.3 (2.7)	70.4 (1.7)
Leaves	Unshaded	82.1 (0.8)	82.3 (0.7)	83.9 (1.4)	83.6 (0.6)
Leaves	Shaded	80.8 (0.7)	80.4 (1.3)	83.1 (0.6)	81.6 (1.2)

Full names of the chemical compounds are given in Table 3.

**Table 3.** (**a**) Standardized discriminant coefficients of individual phenolic concentrations in the bark of *F. sylvatica* in the three discriminant axis of Figure 4. (**b**) Classification matrix of sites in terms of the four retained (most discriminative) phenolics. Row sites are predicted to belong in the column sites. Numbers in parentheses are leave-one-out affiliations.

**a**		**Standardized Discriminant Coefficient**		
	F-statistic*	**CDA 1**	**CDA 2**	**CDA 3**
*cis*-coniferin	25.45	1.65	0.36	−0.38
catechin	42.29	0.52	0.88	0.57
*cis*-isoconiferin	–	–	–	–
*cis*-syringin	20.44	1.64	0.27	−0.46
(2*R*,3*R*)-(+)-glucodistylin	107.85	−0.30	−0.96	0.71
(2*S*,3*S*)-(–)-glucodistylin	–	–	–	–
(2*R*,3*R*)-taxifolin-3-D-β-xylopyranosid	30.80	0.59	0.28	−0.81

**b**	**Aghioneri_F**	**Aghioneri_MD**	**Bellavoda_MC**	**Fracto_FQ**	**% Correct**
Aghioneri_*F*	5 (4)	0	0	0 (1)	100 (80)
Aghioneri_*MD*	0	5	0	0	100
Bellavoda_*MC*	0	0 (1)	5 (4)	0	100 (80)
Fracto_*FQ*	0	0	0	5	100
Total	5 (4)	5 (6)	5 (4)	5 (6)	100 (90)

* All values are significant at the level 10^−4^.
